# Age at menarche and risk of gestational diabetes mellitus: a population-based study in Xiamen, China

**DOI:** 10.1186/s12884-019-2287-6

**Published:** 2019-04-25

**Authors:** Liying Wang, Bing Yan, Xiulin Shi, Haiqu Song, Weijuan Su, Bingkun Huang, Yuxian Zhang, Shunhua Wang, Fuping Lv, Mingzhu Lin, Xuejun Li

**Affiliations:** 1 Xiamen Diabetes Institute, Xiamen, China; 2 Department of Endocrinology and Diabetes, The First Affiliated Hospital, Xiamen University, No.55 Zhenhai Road, Xaimen, 361003 China

**Keywords:** Body mass index, Gestational diabetes mellitus, Menarche, Pregnancy, Woman

## Abstract

**Background:**

It has been reported that earlier age at menarche is associated with a higher risk for type 2 diabetes mellitus. However, the relationship between age at menarche and gestational diabetes mellitus is inconsistent across studies. We hypothesized that an earlier age at menarche would predict the gestational diabetes mellitus risk.

**Methods:**

This was a population-based, retrospective cohort study of 70,041 women aged 18 to 53 years old, conducted between 2011 and 2018. The subjects were recruited from the Medical Birth Registry in Xiamen, China. Age at menarche was categorized as 8–12, 13, 14, 15, 16–19 years old. Logistic regression analysis and spline analysis was used to assess the risk of the menarche age group for gestational diabetes mellitus. Linear regression analysis was performed to evaluate independent associations between age at menarche and fasting plasma glucose and blood glucose 1 hour and 2 hours after a 75-g of glucose load between 24 and 28 weeks’ gestation.

**Results:**

The overall prevalence of GDM was 17.6%. After adjustment for family history of diabetes, earlier age at menarche (8–12, and 13 years old) was associated with increased odds for GDM (OR, 1.08; 95% CI, 1.02–1.15, and OR, 1.07; 95% CI, 1.03–1.14, respectively) compared with average age at menarche (14 years old). With further adjustment for pre-pregnancy body mass index, blood pressure, educational level, age at delivery, and hepatitis B surface antigen status, this association was attenuated (OR, 0.93, and OR, 1.02, respectively). Multivariable-adjusted spline regression models showed a linear dose-response association between age at menarche and GDM (P for nonlinearity, 0.203; P for linearity, 0.006). On linear regression analysis, earlier age at menarche was significantly associated with increased blood glucose one and 2 hours after a glucose load but not with the fasting plasma glucose.

**Conclusions:**

As expected, early age at menarche was found to be associated with an increased risk of gestational diabetes mellitus. However, this association may be mediated by potential confounding factors other than age. An additional finding was that earlier menarche was significantly related with elevated pregnancy glucose concentrations after a glucose load.

## Background

Gestational diabetes mellitus (GDM) is one of the most common pregnancy complications, affecting 9.3–19.7% of all pregnancies in China [1–3]. With the increasing prevalence of GDM and given the strong link between GDM and type 2 diabetes, the identification of women who are at high risk of GDM has important public health and clinical significance. Menarche is an indicator of pubertal onset and the beginning of reproductive life in women. Studies have demonstrated an association between an earlier age at menarche and elevated fasting glucose, fasting insulin, insulin resistance, and the risk for type 2 diabetes [4–7], metabolic syndrome [8], all cause or cardiovascular disease mortality [9, 10], and breast cancer [11]. The precise mechanism by which early age at menarche may increase the risk of GDM is not clear. Some studies indicated that the early age at menarche was a risk factor of GDM [12–14]. Additionally, Early age at menarche has been associated with risk factors for GDM such as excessive pre-pregnancy obesity [15–17], insulin resistance [18], and hormonal changes [19, 20]. However, the potential relationship of age at menarche and the risk of GDM have been inconclusive. Some studies founded a potential association [14, 21, 22], whereas others show no association [23]. In this study, we examined whether there was an independent association between age at menarche and GDM risk, or with raised pregnancy glucose concentrations, in Chinese women.

## Methods

### Study design and population

This study was a retrospective evaluation of the association between age at menarche and GDM risk. The data were collected between January 2011 and March 2018 from the Medical Birth Registry of Xiamen (MBRX), China. The MBRX was established in 2007 that is based on compulsory notification of all live and stillbirths from 12 weeks’ gestation. The MBRX contains information on maternal characteristics (age at menarche, maternal age, education, body mass index [BMI], obstetric history, etc.); pregnancy, labor, and delivery characteristics (gestational diabetes, gestational weight gain, gestational age at delivery, hypertension in pregnancy, etc.); and birth outcomes (fetal and neonatal death, birth weight, Apgar score at 5 min, etc.).

The population in this cohort study was derived from the MBRX that was linked by individual record linkages to the Xiamen citizen health information system using the person-unique identification number assigned to each Xiamen citizen. All the study participants were over 18 years of age. Between 24 and 28 weeks’ gestation, the mothers without diagnosed diabetes were advised to undergo a 75-g oral glucose tolerance test (OGTT) after fasting overnight. Participants were excluded who were known to have a multiple pregnancy or had received medical treatment for chronic diseases, such as oral glucocorticoids, thiazide diuretics, β-blockers, ACE inhibitors, or antiretroviral agents, before OGTT. From 2011 to 2018, there were 279,992 births recorded in this Register system. We restricted our cohort to 70,041 singletons born to pregnant women who underwent a 75-g OGTT and had menarche age records between March 1, 2011 and March 30, 2018. Study procedures were approved by the institutional review boards of the First Affiliated Hospital of Xiamen University, Xiamen, China.

### GDM assessment

GDM cases were identified with the OGTT that conducted between 24 and 28 weeks’ gestation, which is considered the optimal period to make this diagnosis. According to the 2014 National Health and Family Planning Commission of the People’s Republic of China criteria, pregnant women were considered to have GDM if one of the following plasma glucose values was met or exceeded: 0 h, 5.1 mmol/L; 1 h, 10.0 mmol/L; or 2 h, 8.5 mmol/L, after a 75-g glucose load. Even if the test was performed after 28 weeks, it was considered valid.

### Age at menarche

This was defined as the age at the first menstrual period. The information on age at menarche was obtained by questionnaire: “At what age did you have your first menstrual period?”. The MBRX summarized the data that came from the Women and Children Medical and Healthcare Centre of Xiamen. For our analysis, the age at menarche of participants was divided as: 8–12 years old, 13 years old, 14 years old, 15 years old, and 16–19 years old. The early age at menarche (< 13 years) that consistent with the categories used in other research [24].

### Statistical analysis

Analyses were restricted to the 70,041 women who recorded their menarche at a physiological age between 10 and 20 years. Continuous variables were expressed as means and standard deviations, whereas categorical variables were expressed as percentages. One way analysis of variance (ANOVA) was used to compare the means of the continuous variables, and the Chi-squared test was used to compare the proportions of the categorical variables. Logistic regression analysis was performed to estimate the odds ratios (OR) and 95% confidence interval (CI) of GDM for each age at menarche group (8–12, 13, 14, 15, and 16–19 years old), with the median age at menarche (14 years) serving as the reference category, after adjusting for potential confounding and mediating factors. Linear regression analysis was conducted to evaluate independent associations between the age at menarche and fasting plasma glucose, one and 2 hours after a 75-g glucose load between 24 and 28 weeks’ gestation. Significance tests were two-tailed and a *P*-value < 0.05 was considered statistically significant. The data analysis for this article was generated using SPSS (version 19 of the SPSS system for the Windows × 64-based system) and SAS software (version 9.4, SAS Institute Inc. Cary, NC, USA.).

## Results

Two hundred nine thousand nine hundred fifty-one participants were excluded, 70,041 participants were included. By comparing the main characteristics, we found no difference between the included and excluded population. The specific characteristics of the excluded and included groups are as following: age: 28.4 ± 4.8 vs. 28.4 ± 4.1, *P* = 0.291; BMI: 21.1 ± 3.0 vs. 21.0 ± 2.9, *P* = 0.236; plasma glucose, 4.5 ± 0.5 vs. 4.5 ± 0.4, *P* = 0.342; Systolic blood pressure, 107.6 ± 10.4 vs. 107.5 ± 10.7, *P* = 0.443; Diastolic blood pressure, 65.7 ± 7.8 vs. 65.6 ± 7.9, *P* = 0.551; Education (≤ 9 years), 24.1% vs. 23.5%, *P* = 0.360; Family history of diabetes, 2.7% vs. 2.6%, *P* = 0.523; Family history of hypertension, 6.4% vs. 6.4%, *P* = 0.481. The pregnant women included in our analysis had a mean (SD) age at menarche of 14.1 (1.6) years. The proportion of participants reporting menarche at ages at 8–12, 13, 14, 15, and 16–19 years old was 10.4, 24.6, 30.0, 19.5, and 15.5%, respectively. The characteristics and mean 75-g oral glucose tolerance test plasma glucose levels of the participants at 24 to 28 weeks’ gestation (based on age at menarche) are presented in Table 1.

**Table 1:** Clinical characteristics of subjects by age at menarche

Age at menarche (year)	8–12 years (*n* = 7770, 10.4%)	13 years (*n* = 18,419, 24.6%)	14 years (*n* = 22,585, 30.0%)	15 years (*n* = 14,650, 19.5%)	16–19 years (*n* = 11,635, 15.5%)	*P*-value
Age, year	28.5 ± 4.0	28.3 ± 4.0	28.3 ± 4.0	28.4 ± 4.2	28.6 ± 4.3	< 0.001*
Body mass index, BMI	21.7 ± 3.0	21.2 ± 3.0	20.9 ± 2.8	20.9 ± 2.8	20.6 ± 2.7	< 0.001*
Systolic blood pressure (mmHg)	108.3 ± 10.6	107.9 ± 10.6	107.4 ± 10.6	107.5 ± 10.6	107.3 ± 10.6	< 0.001*
Diastolic blood pressure (mmHg)	66.4 ± 7.9	65.9 ± 7.9	65.5 ± 7.9	65.3 ± 7.8	65.1 ± 7.8	< 0.001*
Education (≤ 9 years)	937(13.5%)	3377(20.6)%	4271(23.7)%	3744(29.1%)	3601(34.9%)	< 0.001*
Family history of diabetes	333(4.3%)	565(3.1%)	544(2.4%)	323(2.2%)	201(1.7%)	< 0.001*
Family history of hypertension	586(7.5%)	1112(6.0%)	1168(5.2%)	757(5.1%)	519(4.5%)	< 0.001*
HbsAg positive	222(6.4%)	785(8.5)%	874(8.2%)	672(9.1%)	1191(9.2%)	< 0.001*
Glucose 0 min OGTT (mmol/L)	4.51 ± 0.4	4.50 ± 0.4	4.49 ± 0.4	4.48 ± 0.39	4.48 ± 0.39	< 0.001*
Glucose 60 min OGTT (mmol/L)	7.91 ± 1.65	7.85 ± 1.69	7.81 ± 1.68	7.80 ± 1.67	7.80 ± 1.67	< 0.001*
Glucose 120 min OGTT (mmol/L)	6.78 ± 1.39	6.71 ± 1.38	6.68 ± 1.38	6.67 ± 1.37	6.66 ± 1.38	< 0.001*

*p <  0.05

Compared with earlier age at menarche (8–12 years old), women at a later age at menarche (16–19 years old) had a lower body mass index (20.6 ± 2.7 vs. 21.7 ± 3.0 kg/m^2^, *P* <  0.001), systolic blood pressure (107.3 ± 10.6 vs. 108.3 ± 10.6 mmHg, *P* <  0.001), and diastolic blood pressure (65.1 ± 7.8 vs. 66.4 ± 7.9 mmHg, *P* <  0.001). A higher proportion of women with an earlier age at menarche had a family history of diabetes (4.3% vs. 3.1% vs. 2.4% vs. 2.2% vs. 1.7%, respectively, *P* <  0.001) and family history of hypertension (7.5% vs. 6.0% vs. 5.2% vs. 5.1% vs. 4.5%, respectively, *P* <  0.001) than those with an average or late age at menarche, but a lower proportion had chronic hepatitis B infection (6.4% vs. 8.5% vs. 8.2% vs. 9.1% vs. 9.2%, respectively, *P* <  0.001) (Table 1).The results of the OGTT showed that women with an earlier age at menarche had higher fasting, one-, and two-hour plasma glucose levels than those with average or later age at menarche (all *P* <  0.001) (Table 1).

**Fig. 1:**
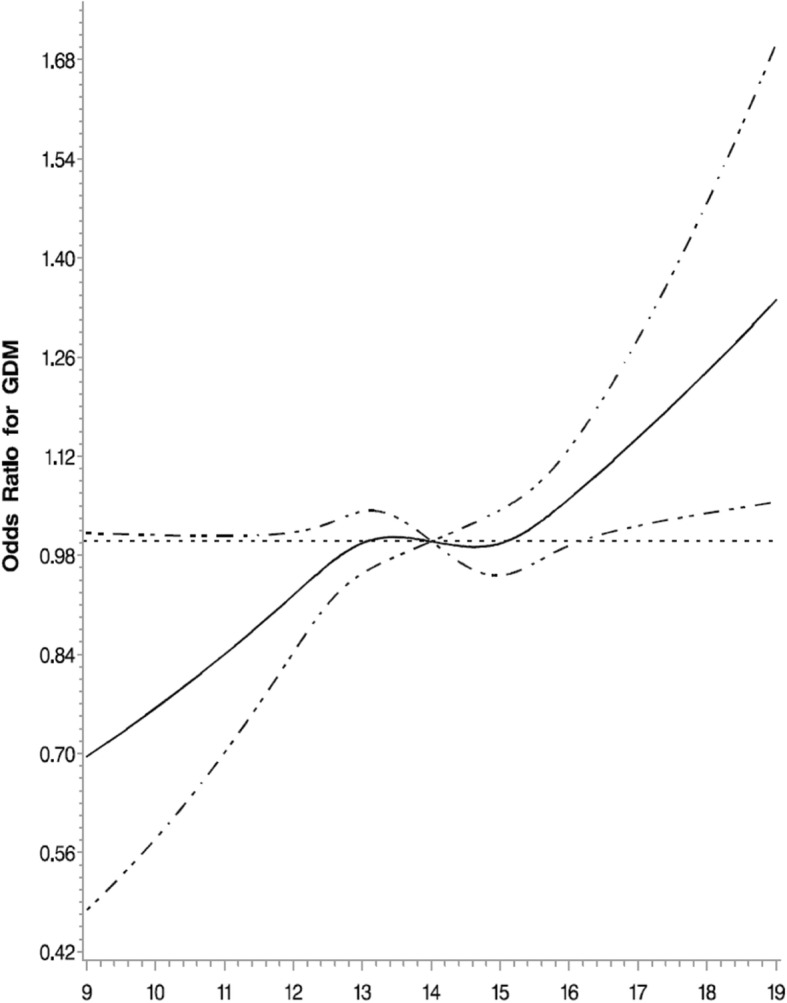
Association of age at menarche and GDM. Odds ratios (ORs) and 95% CIs derived from restricted cubic spline regression. Reference point is 14 years at menarche. ORs were adjusted for the same variables as model 4 in Table 2

In the present study, the overall prevalence of gestational diabetes mellitus was 17.6%. The GDM prevalence, according to the age at menarche, was 10.4% (8–12 years old), 24.6% (13 years old), 30.0% (14 years old), 19.5% (15 years old), and 15.5% (16–19 years old). The crude GDM prevalence was highest in those with an earlier age at menarche. Table 2 presents the odds ratio (OR) for GDM by age at menarche. After adjustment for family history of diabetes, earlier age at menarche (8–12 years old) was associated with increased odds for GDM (OR 1.08 [95% CI 1.02–1.15]) compared with average age at menarche (14 years old). The association was no longer significant (OR 0.93 [95% CI 0.85–1.03]) after additional adjustment for pre-pregnancy body mass index, blood pressure, educational level, age at delivery, and hepatitis B surface antigen (HBsAg) status. Furthermore, Age at menarche menopause was treated as a continuous variable, the results showed that the crude OR for GDM per 1 year older at menarche was 0.98 (95% CI 0.96, 0.99), however, these associations attenuated towards the null following adjustment for blood pressure and BMI (Table 3). In addition, multivariable-adjusted spline regression models showed a linear dose-response association between age at menarche and GDM (P for nonlinearity, 0.203; P for linearity, 0.006) (Fig. 1)*.* Meanwhile*,* linear regression analysis (Table 4) showed an inverse association between the age at menarche and fasting, one- and two-hour glucose levels. The relationship with fasting glucose was no longer significant after adjustment for family history of diabetes, education levels, age at delivery, chronic hepatitis B virus status, blood pressure, and pre-pregnancy BMI. Even after adjusting for possible confounding variables, age at menarche was significant and inversely associated with the one- and two-hour glucose levels.

**Table 2:** Adjusted odds ratios (ORs) with associated 95% confidence interval (CI) of gestational diabetes mellitus

	Age at menarche										
	8–12 years (*n* = 7770)		13 years (*n* = 18,419)		14 years (*n* = 22,585)		15 years (*n* = 26,285)		16 -19 years (*n* = 26,285)		*P*_trend_
	OR	95% CI	OR	95% CI	OR	95% CI	OR	95% CI	OR	95% CI	
Crude model	1.09	1.02–1.16	1.08	1.02–1.13	1	referent	0.97	0.92–1.02	0.99	0.93–1.05	0.001
Model 1	1.08	1.02–1.15	1.07	1.03–1.14	1	referent	0.97	0.92–1.02	0.99	0.94–1.05	0.001
Model 2	1.08	1.01–1.16	1.10	1.05–1.17	1	referent	0.97	0.92–1.02	0.98	0.92–1.04	0.001
Model 3	1.03	0.95–1.13	1.07	0.99–1.14	1	referent	0.96	0.89–1.03	1.02	0.95–1.10	0.113
Model 4	0.93	0.85–1.03	1.02	0.95–1.08	1	referent	0.97	0.91–1.05	1.06	0.99–1.18	0.094

Model 1 adjusted for family history of diabetes

Model 2 adjusted for variables in model 1 plus education levels, age at delivery, chronic hepatitis B virus status

Model 3 adjusted for variables in model 2 plus systolic blood pressure, diastolic blood pressure

Model 4 adjusted for variables in model 3 plus pre-pregnancy BMI

**Table 3:** Multivariable logistic of regression of gestational diabetes mellitus (Age at menarche as a continuous variable)

Age at menarche (year)	OR	95% CI	*P*-value
Crude model	0.975	0.962–0.989	< 0.001
Model 1	0.979	0.966–0.993	< 0.001
Model 2	0.970	0.956–0.984	< 0.001
Model 3	0.987	0.970–1.005	0.1627
Model 4	1.002	1.000–1.041	0.0610

Model 1 adjusted for family history of diabetes

Model 2 adjusted for variables in model 1 plus education levels, age at delivery, chronic hepatitis B virus status

Model 3 adjusted for variables in model 2 plus systolic blood pressure, diastolic blood pressure

Model 4 adjusted for variables in model 3 plus pre-pregnancy BMI

**Table 4:** Multivariable linear regression of plasma glucose of OGTT

Age at menarche (year)	Coefficient	SE	*P*-value
Glucose 0 min OGTT (mmol/L)			
Model 1	- 0.004	0.001	< 0.001
Model 2	−0.009	0.002	< 0.001
Model 3	−0.006	0.002	< 0.001
Model 4	0.004	0.002	0.059
Glucose 60 min OGTT (mmol/L)			
Model 1	−0.030	0.002	< 0.001
Model 2	−0.030	0.004	< 0.001
Model 3	−0.020	0.008	0.021
Model 4	−0.016	0.004	0.040
Glucose 120 min OGTT (mmol/L)			
Model 1	−0.030	0.005	< 0.001
Model 2	−0.032	0.005	< 0.001
Model 3	−0.023	0.007	< 0.001
Model 4	−0.021	0.005	0.003

Model 1 adjusted for family history of diabetes

Model 2 adjusted for variables in model 1 plus education levels, age at delivery, chronic hepatitis B virus status

Model 3 adjusted for variables in model 2 plus systolic blood pressure, diastolic blood pressure

Model 4 adjusted for variables in model 3 plus pre-pregnancy BMI

## Discussion

We observed two major findings in this study. On the one hand, earlier menarche tended to be associated with gestational diabetes mellitus after adjusting for age, but after further adjustment for pre-pregnancy body mass index, blood pressure, educational level, family history of diabetes, and hepatitis B surface antigen (HBsAg), this association was attenuated. Of which, many studies indicated a significant association between HBsAg positive and GDM [25–27]. Therefore, we adjusted the HBsAg factor that resulted in the association was weak in this study. Meanwhile, later age at menarche may be not related with gestational diabetes mellitus. On the other hand, earlier age at menarche was significantly associated with increased one- and two-hour blood glucose after a glucose load at 24 to 28 weeks’ gestation, even after adjusting for a number of confounding factors. The association may be mediated through age, blood pressure, and pre-pregnancy BMI between age at menarche and fasting plasma glucose at 24 to 28 weeks’ gestation. To our knowledge, there are a few studies demonstrating a significant association between an earlier age at menarche and elevated glucose levels after a glucose load in China.

Meanwhile, we found that the prevalence of GDM (17.6%) is higher than other populations in this study. One research reported that the total incidence of GDM in mainland of China was 14.8% [28]. A vast territory and a large population in China with significant differences in ethnicities, diets, lifestyles, and regions, and these factors may result in differences in prevalence of GDM reported in various regions. In addition, our study indicated that the average age at menarche (14.1 ± 1.6) is higher in this population than in many of the published studies. A study showed the overall mean age at menarche was 12.7 years in Korean girls [24]. Besides, another Chinese study expressed that mean age at menarche was 13.1 ± 1.2 years in Wuhan, a city located in Hubei province, China [22]. A large population in China with significant differences in ethnicities, diets, and lifestyles may lead to differences in mean age at menarche reported in various regions. As well, the BMI of categories in this research was lower than a U.S. study [29]. L.W. Chen et al., indicated that mean age at menarche at 13 years, the BMI of pre-pregnancy (23.3 ± 4.1 kg/m^2^) was higher than our study (21.3 ± 3.0 kg/m^2^). This significant difference was original from the difference of ethnics and smaller sample size compared with other study.

Whereas most studies have reported an increased risk of type 2 diabetes with earlier age at menarche, few studies have examined a possible association between age at menarche and the risk of GDM. These few studies have shown inconsistent results; most studies found that earlier age at menarche is associated with an increased risk of GDM [13, 14, 22, 29]. In addition, a meta-analysis showed that women with menarche at an early age (≤ 11 years) had a higher GDM risk with no significant heterogeneity between studies (*P* = 0.17; I^2^ = 38) [13]. Whereas Dishi et al. found no such association [23]. Dishi et al. also suggested that an association of early menarche with the risk of GDM could differ that affected by pre-pregnancy BMI. In our study, this association was independent of age at delivery, but the association was significantly disappeared after further adjustment for the pre-pregnancy BMI. Both increased BMI and blood pressures are likely to be associated with insulin resistance in pregnancy which has also been shown to be negatively associated with age at menarche. However, our further research showed that the results of unadjusted variable of BMI or BMI and blood pressure that indicated the earlier age at menarche was significantly associated with GDM.

Few studies have demonstrated that early age at menarche is linked to higher fasting, one-, and two-hour plasma glucose levels, even after adjustment for confounding factors [22, 30, 31]. However, our results only showed a significant association existed between earlier age at menarche and stimulated glucose levels. The association was completely mediated by age at delivery, pre-pregnancy BMI, and blood pressure levels between age at menarche and fasting plasma glucose in pregnancy. Interestingly, our study indicated that the OGTT 60 min and 120 min glucose concentrations were higher at earlier age at menarche than mean or later age at menarche. It is noteworthy that multivariable linear regression showed that the coefficient was very small for association between glucose concentrations and age at menarche, despite the statistically significant *p*-values due to the large sample size. Studies reported that earlier age at menarche might be associated with adulthood higher estrogen levels and lower serum sex hormone-binging globulin levels among women [19], which could lead to higher OGTT 60 min and 120 min glucose levels. However, the KORA F4 study founded that young age at menarche was significantly related to higher fasting plasma glucose, and 120 min glucose concentrations [30]. Meanwhile, one published study showed a negative linear association between age at menarche and OGTT 60 min glucose concentrations in pregnancy [22]. Besides, a research elucidated that there was no significant association existed between menarche age and diabetes [32]. The sample sizes of populations and methods of statistical analysis may lead to aforementioned differences.

The strengths of our study included the large sample size and the GDM was diagnosed based on the oral glucose tolerance test. Nevertheless, several limitations also need to be acknowledged. Firstly, we were unable to obtain adequate information on childhood risk factors, which could influence both age at menarche and abnormal glucose metabolism. Secondly, because age at menarche was self-reported, misclassification may have occurred. Thirdly, this study was restricted to a medium-sized city in China with a relatively developed economy. However, data on income or job type was not collected. Therefore, the profile of socioeconomic status of the study population was not clearly known. And the results may not be generalizable to other socioeconomic groups. Fourthly, the results of this study were only applicable to Asian populations. Finally, it is inevitable that the linked and registered data are a potential source of bias in results of researches, yet researchers often discover it difficult to evaluate the extent of bias, because of the separation of linkage and analysis processes. Besides, we did not measure biomarkers for the mechanistic assessment of any possible relationships.

Taken together, this association between age at menarche and GDM should be further investigated in other study populations to identify our observations. Furthermore, the mechanistic researches are needed that elucidate underlying pathophysiologic mechanisms of observed correlation may help confirm therapeutic targets of a disorder related to significant offspring mortality. In addition, association between early age at menarche and GDM may help confirm women at high risk of GDM and adopt early prevention strategies.

## Conclusions

In summary, we found an expected association between early age at menarche and increased GDM risk, but this association was less substantial when controlling for certain confounding factors. One significant additional finding from this study was that earlier menarche was inversely associated with pregnancy glucose concentrations following a glucose load. However, the coefficients for the outcomes were small. Future studies are needed to confirm our observations and/or expand on our results in other populations. Improved understanding of the relationship between the age at menarche and the risk of GDM will facilitate prevention and early detection.

## Abbreviations

75-g OGTT: 75-g oral glucose tolerance test
BMI: Body mass index
FPG: Fasting plasma glucose
GDM: Gestational diabetes mellitus
IADPSG: the International Association of Diabetes and Pregnancy Study Groups
IDF: International Diabetes Federation
MBRX: Medical Birth Registry of Xiamen
